# The Effects of Rainfall Events on the Composition and Diversity of Microplastics on Beaches in Xiamen City on a Short-Term Scale

**DOI:** 10.3390/toxics12050375

**Published:** 2024-05-20

**Authors:** Xueyan Li, Fengrun Wu, Chengyi Zhang, Tao Wang

**Affiliations:** 1 School of Environmental Science and Engineering, Xiamen University of Technology, Xiamen 361024, China; 15235771532@163.com (X.L.); zhangchengyi1001@163.com (C.Z.); 2 State Key Laboratory of Estuarine and Coastal Research, East China Normal University, Shanghai 200241, China; warriortao723@gmail.com

**Keywords:** microplastic pollution, precipitation, coastal zone, cumulative effect

## Abstract

Coastal beaches are vulnerable to microplastic pollution originating primarily from terrestrial and marine sources or the in situ weathering of plastic waste. The present study investigates the effects of rainfall events on the composition and diversity of microplastics on beaches in Xiamen City on a short-term scale. In the results, the quantity of microplastics in beach sediments was 245.83 ± 11.61 items·kg^−1^ (mean ± standard error). The abundance of microplastics did not differ after each rainfall event but significantly decreased after multiple rainfall events. When the diversity of microplastics in the coastal area was evaluated, the Shannon-Wiener index and Pielou’s index also decreased from 3.12 and 0.64 to 2.99 and 0.62, respectively, after multiple rainfall events. Rainfall had varying effects on microplastics depending on their size and shape, with particles smaller than 500 μm experiencing pronounced reductions. There was a significant negative correlation between the abundance of microplastics and the grain size of sand, but a positive correlation with sediment moisture content. We encourage the consideration of the potential impact of rainfall events during sample collection to ensure the reliability of the data. We also recommend using diversity indexes to help in understanding the influence of physical processes on microplastic distribution and their mechanisms.

## 1. Introduction

In the present era, plastic pollution has become a significant global concern. Since their inception in the 1860s, plastics have found extensive application in various industries, including the industrial, agricultural, and medical sectors [1]. This widespread usage can be attributed to its advantageous properties, such as its low density, low thermal and electric conductivity, and corrosion resistance [2]. Over the last two decades, improper management of plastics has severely affected global ecosystems [3]. It is estimated that approximately 1.2 billion tons of plastic waste will be directed toward landfills or infiltrate the natural environment by 2050 [4]. Plastic materials less than 5 mm in size are classified as microplastics [5]. These particles can originate from deliberate manufacturing processes (primary) or the physical and chemical breakdown of larger plastic items in the environment (secondary) [6].

Numerous studies have extensively examined the occurrence of microplastics in diverse ecosystems, revealing their presence in freshwater systems [7], marine habitats [8], deep-sea environments [9], and beach sediments [10]. Microplastics have also been detected in various organisms, including zooplankton [11,12], crabs [13,14], fish [15,16], and mussels [17,18]. Additionally, microplastics can adsorb surrounding pollutants onto their surfaces and deliver them to humans through food webs, raising considerable concern [19,20,21].

Coastal beaches are located in the transitional zone between marine and terrestrial domains. They provide many essential services, including the prevention of wave erosion, water purification, and habitats for organisms [22,23,24]. However, owing to recreational and tourism development, coastal beaches are vulnerable to the entrapment of microplastic debris [24]. Previous studies detected a wide distribution of microplastics in beach sediments, water columns, and organisms [23,25,26]. These particles are mainly derived from terrestrial and marine sources via river transport and tidal deposition [27,28,29]. Moreover, microplastics that arise from the in situ weathering of plastic waste can also accumulate on coastal beaches [30]. 

Understanding the factors that regulate the distribution of microplastics on beaches is crucial for effective coastal environmental management. In terms of the temporal scale, microplastic contamination has been shown to vary across seasons. Some studies have suggested that more microplastics can be detected on beaches in rainy seasons than in dry seasons due to the input of microplastics from overland runoff [31,32]. In contrast, other studies have reported lower microplastic contamination during rainy seasons, which is attributed to scouring effects on beaches during rainfall events [33,34]. These studies conducted on seasonal scales suggest that precipitation significantly influences the composition of microplastic pollution on coastal beaches. However, the mechanisms by which single rainfall events influence microplastics have not been clearly defined. Few studies have focused on the effects of rainfall events on microplastic composition on a short-term scale. In particular, it is crucial to comprehend whether there is a notable variation in the composition of microplastics on beaches following an individual rainfall event during the rainy season or if there is a cumulative process at play. This understanding holds significant importance in simulating the migration processes of microplastic pollution in coastal regions. Regarding heterogeneity on a spatial scale, the characteristics of the substrate environment, such as the particle size of sand and moisture content, have also been reported to influence the migration of microplastics [35,36]. Therefore, when examining the influence of rainfall on microplastics on a short-term scale, it is necessary to consider the characteristics of microplastics and the combined effects of the substrate environment.

Moreover, specific characteristics of microplastics often indicate the sources of microplastics. For example, previous studies have indicated that most fibrous particles in coastal areas come from fibers generated from the washing of clothes carried by upstream river runoff [28]. Fragments often originate from the breaking process of large plastic debris on beaches [37]. If the assemblage of microplastics in the coastal area is viewed as a biological community, its variation in diversity may reflect the changes in potential sources of pollution [38,39]. Thus, it could be helpful to use diversity indexes to reveal the effects of rainfall events on the distribution of microplastics and their corresponding mechanisms. 

To bridge this knowledge gap, we investigated microplastics on five coastal beaches in Xiamen City, Fujian Province, China. Based on existing research, we hypothesized that (1) an individual rainfall event has a minimal influence on the abundance of microplastics, though the impact may be amplified after multiple rainfall events; (2) microplastics of different sizes and shapes may respond differently to rainfall events, resulting in a fluctuation in their diversity on a short-term scale; and (3) microplastic composition can exhibit different patterns of variation in diverse substrate environments during rainfall events.

## 2. Materials and Methods

### 2.1. Study Sites and Sample Collection

**Figure 1:**
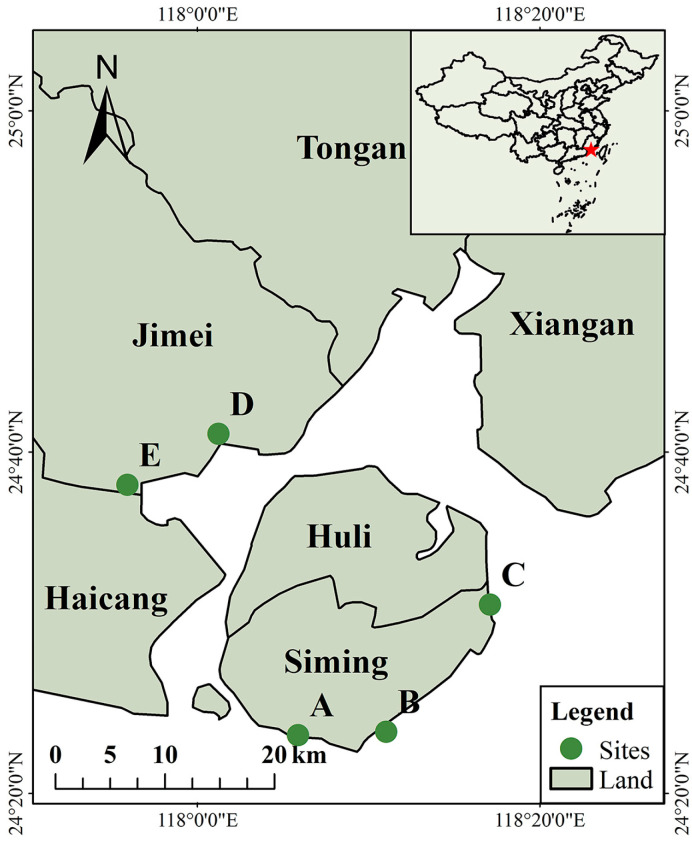
Geographical locations of study sites in Xiamen City, China.

Our study was conducted in Xiamen City, Fujian Province, China, within the longitude and latitude ranges from 117°52’56’’ E to 118°22’26’’ E and from 24°23’13’’ N to 24°54’26’’ N, respectively (Figure 1). It consists of six administrative divisions: Huli District, Siming District, Jimei District, Xiang’an District, Tong’an District, and Haicang District. The tidal patterns exhibit regular semidiurnal fluctuations and an average tidal range of 4.08 m [40]. The area experiences a subtropical maritime monsoon climate, characterized by an average annual temperature and rainfall of 21 ℃ and 1200 mm, respectively [41,42]. The rainy season in Xiamen extends from March to September and is mainly influenced by the southeast and southwest monsoons. During this period, precipitation is frequent, often accompanied by brief showers or continuous drizzle. Land scouring is highly likely under this rainfall pattern.

In this study, we established fixed sampling sites on five beaches in Xiamen City, including Baicheng (A), Huangcuo (B), Guanyinshan (C), Xinglinwan (D), and Lehai (E). All chosen beaches are recreational, subjected to various human activities such as tourism and beachcombing. No noticeable cleaning activities are observed at these sites. They are strategically positioned in different orientations throughout the city, displaying a wide range of substrate characteristics (Figure 1). At each site, we marked ten sampling plots parallel to the coastline. All plots were positioned at the high tide line. The distance between the plots was approximately 20 m.

We collected sediment samples from the ten plots at each beach prior to (PR) and after (AR) the three continuous rainfall events during the rainy season of 2023. These ten samples were considered replicates for each sampling site. Three continuous rainfall events were characterized by different durations and rainfall intensities (Table 1). They occurred within a 50-day timescale, which helped to minimize the interference from factors such as cleaning activities on the composition of microplastics. An independent rainfall event was identified when there was no precipitation for a sustained 24 h period. We collected rainfall duration and intensity data from the Xihe Energy Weather Big Data Platform (www.xihe-energy.com, accessed on 17 November 2023). All three sampling events were specifically conducted during neap tidal periods, which conveniently helps to minimize the potential impact of hydrodynamics on the data [29].

In the field, a stainless-steel split-tube sampler was used to obtain sediment cores from each plot at a low tide. The cores had a depth and diameter of 10 and 5 cm, respectively. Subsequently, the sediment samples were carefully transferred to clean cylindrical iron boxes and transported to the laboratory. During the sampling period, the pore-water salinity was measured using a soil moisture detector (Zhengzhou Hongchuang Environmental Protection Technology Co., Ltd. HC-S400, Zhengzhou, China).

### 2.2. Laboratory Processing

Upon arrival at the laboratory, the wet weight of each sediment sample was measured. Subsequently, all samples were dried in an oven set at 75 °C for at least 48 h until a constant weight was reached. The dried samples were weighed to an accuracy of 0.1 g. The moisture contents of the sediment samples were determined based on these weights. The bulk density of the sediments was calculated from the division of the dry weight by the volume of the sediment core. The median grain size of the sediments was measured through sieving.

Microplastics were extracted from the sediments using the density separation method [5,28]. Following thorough mixing, a subsample of 20 g of sediment was taken from each of the dried samples and placed into separate glass beakers (N = 300). To degrade the organic matter, 20 mL of 30% H_2_O_2_ (Sinopharm Chemical Reagent Co., Ltd. Shanghai, China) was added and allowed to react for 24 h at room temperature [14,43]. We then added 200 mL of a filtered, saturated NaCl solution (1.2 g·cm^−3^) to the beaker and stirred the mixture with a glass rod for 2 min. After settling for 24 h, the liquid supernatant was vacuum-filtered through a glass filter paper of 1 μm pore size.

We visually counted and assessed microplastics on the glass filter paper using a stereomicroscope at 10–20× magnification (Hanguang Optical (Wuxi) Co., Ltd. SZM7045T, Wuxi, China). These identifiable microplastics were classified according to their shapes (fiber, fragment, foam, and film) and colors (black, blue, red, green, transparent, yellow, gray, and white). Additionally, we used a micrometer under the microscope to measure the size (maximum dimension) of each particle. To confirm that the particles that we counted were actually made of plastic, we identified the polymer type of 200 representative particles (100 fibers, 80 fragments, 15 films, and 5 foams) using a Micro Fourier Transform Infrared Spectrometer (Thermo Scientific Nicolet iN10 MX, Waltham, MA, USA) under the transmittance mode. These selected particles are samples collected from various beaches and sampling events. All spectra were post-processed under an automatic baseline correction mode using the OMNICTMPictaTM software (Version 1.5).

### 2.3. Quality Assessment and Quality Control

Field sampling was swiftly conducted to minimize the risk of secondary contamination. The researchers strictly followed rigorous laboratory protocols, wearing cotton laboratory coats and gloves, and avoided using plastic materials. The containers were thoroughly cleaned with pure filtered water prior to use. All solutions used in the experiments underwent double filtration. Three procedural blanks were employed to ensure that there was no contamination from the laboratory setting. The extraction, cleanup, and storage procedures mentioned above were run on three membrane filters without samples. No microplastic particles were identified in the blank samples during this procedure.

### 2.4. Statistical Analysis

The abundance of microplastics in the sediments was calculated as the number of microplastics per kilogram of sediment (n·kg^−1^). IBM SPSS Statistics v.22 was used to analyze data variation. We conducted the Shapiro–Wilk test (two-tailed, *p* < 0.05) to examine the normality of the datasets. To investigate the temporal and spatial variations in the microplastic composition and the physicochemical properties of the sediments, respectively, we performed the Kruskal–Wallis test, followed by pairwise comparisons. A significance level (*p*-value) of 0.05 was employed for all statistical analyses. In the initial stage, to reduce the interference of sites on the results, five sites were considered parallel groups to analyze the variation in the composition of microplastics on beaches caused by rainfall events. Subsequently, we tested the variation in the composition of microplastics at different sites, respectively. 

We also analyzed the variation in the microplastics of different characteristics (sizes, shapes, and colors) prior to and after the rainfall. Additionally, microplastics collected from all sites were treated as a single biological community and subdivided based on three criteria: color, shape, and size. The Shannon–Wiener index (*H*’) and Pielou’s index (*J*’) were used to assess the diversity of microplastics during different rainfall events. The Shannon–Wiener index can quantify the level of uncertainty in the composition of assemblages, while Pielou’s index provides an evaluation of the evenness of communities. The index calculations are given by the following:

$$H^{\prime }=-\sum_{i=1}^{S}P_{i}\ln P_{i}$$

$$J^{\prime }=H^{\prime }/\ln S$$

where *S* is the number of categories and *P_i_* is the proportion of categories *i* [44].

We employed the coefficient of variation (CV) to assess the variability in microplastic composition across different sampling events. CV was calculated using the following equation:

CV = (SD/Mean) × 100%

where SD is the standard deviation of microplastic abundance across all samples at each site and mean is the average microplastic abundance at each site.

In addition, Spearman’s correlation analysis was conducted in R studio (version 4.3.2) to examine the correlations between microplastic composition and substrate properties. 

## 3. Results

### 3.1. Microplastic Composition on the Beaches of Xiamen City

**Figure 2:**
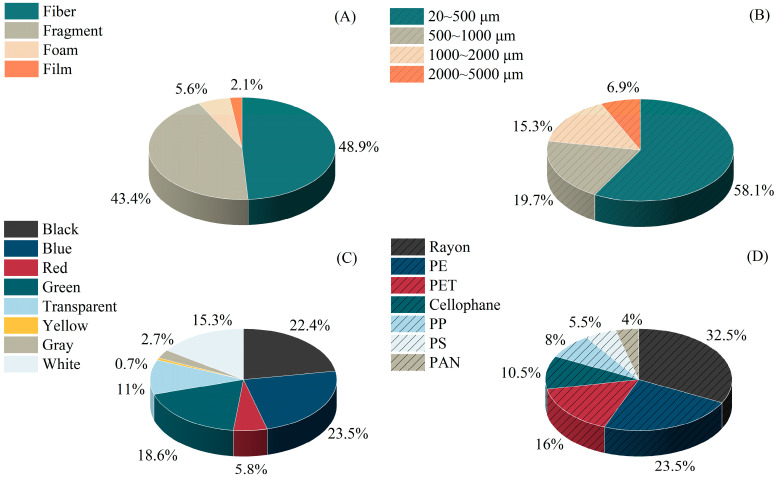
Shape (**A**), size (**B**), color (**C**), and polymer type (**D**) composition (%) of the microplastics on the beaches in Xiamen City.

We detected microplastic particles in 97% of the samples (N = 300). A total of 1475 items were examined, with an average quantity of 245.83 ± 11.61 n·kg^−1^ (mean ± se). Microplastics were categorized into a variety of shapes, including fibers, fragments, foams, and films (Figure S1). Fibers and fragments were the most common, accounting for 48.9% and 43.4%. Foams (5.6%) and films (2.1%) were relatively rare (Figure 2A). Among the different size categories of microplastics, the class of 20–500 μm contributed the highest proportion of 58.1%, followed by the classes of 500–1000 μm and 1000–2000 μm, accounting for 19.7% and 15.3%, respectively. Particles larger than 2000 μm were rare (Figure 2B). Blue and black particles were the most dominant, at 23.5% and 22.4%. The proportion of green particles was also relatively high, with a ratio of 18.6%. White (15.3%), transparent (11%), red (5.8%), gray (2.7%), and yellow (0.7%) accounted for relatively small proportions (Figure 2C). Out of the 200 particles that were detected for polymer types, they contained 7 distinct component types (Figure S2). Rayon accounted for 32.5% of the particles, while 23.5% were identified as polyethylene (PE), and 16.0% were found to be polyethylene terephthalate (PET). The remaining polymer types were not commonly detected, with cellophane, polypropylene (PP), polystyrene (PS), and polyacrylonitrile (PAN) particles present in small proportions (Figure 2D).

### 3.2. Variations in the Abundance of Microplastics Prior to and after Rainfall Events

**Figure 3:**
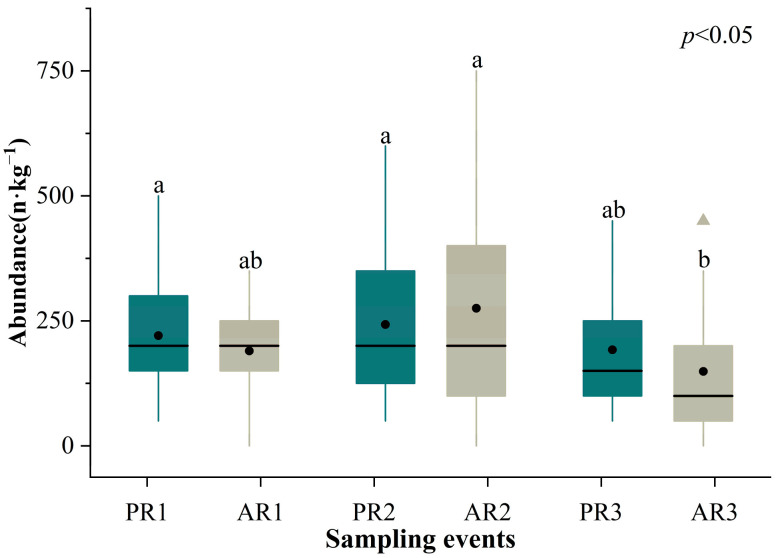
The abundance of microplastics prior to and after three rainfall events. The data are for all samples from five beaches. Note: The lines within boxes indicate medians, the black dots within boxes indicate means, the boxes indicate interquartile intervals, the triangles indicate outliers, and the bars above and below the boxes indicate the upper and lower non-outlier intervals. Different letters represent significant differences among groups at the 95% confidence level.

There were significant temporal variations in the abundance of microplastics among the different sampling events (*p* < 0.05). The abundance decreased significantly in AR3 compared with PR1 (*p* < 0.05), with the lowest value being 100.0 n·kg^−1^. However, the abundance of microplastics before and after all three rainfall events did not differ significantly (Figure 3). 

### 3.3. Variations in the Size Composition of Microplastics Prior to and after Rainfall Events

**Figure 4:**
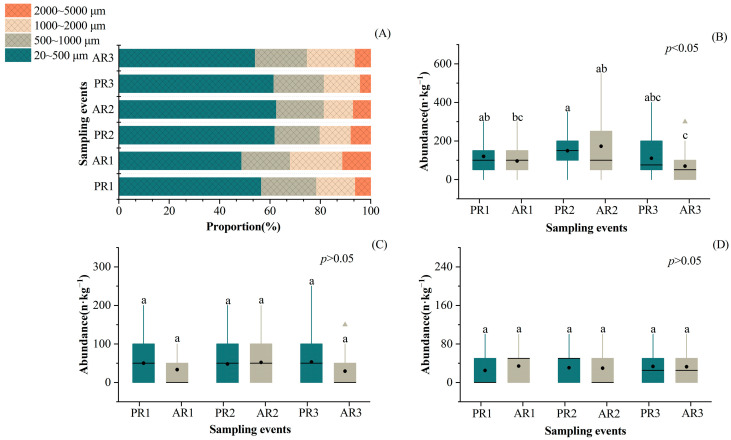
The proportion of microplastics of different sizes (**A**) and the variation in the abundance of microplastics with sizes of 20–500 μm (**B**), 500–1000 μm (**C**), and 1000–2000 μm (**D**) prior to and after three rainfall events. Note: The lines within boxes indicate medians, the black dots within boxes indicate means, the boxes indicate interquartile intervals, the triangles indicate outliers, and the bars above and below the boxes indicate the upper and lower non-outlier intervals. Different letters represent significant differences among groups at the 95% confidence level.

The proportion of microplastics of different sizes varied slightly among the sampling events. Overall, higher percentages of smaller microplastics were observed (Figure 4A). Additionally, microplastics of varying sizes displayed distinct variation patterns during sampling events. The abundance of the 20–500 μm size category differed significantly among the three rainfall events (*p* < 0.05); in particular, contamination was significantly lower in AR3 compared with PR1 (*p* < 0.05) (Figure 4B). However, no significant differences in the abundance of these microplastics were observed before or after each rainfall event. Nevertheless, the abundance of plastics with sizes of 500–1000 μm and 1000–2000 μm was not significantly affected by the three rainfall events (*p* > 0.05) (Figure 4C,D).

### 3.4. Variation in Shape Composition of Microplastics Prior to and after Rainfall Events

**Figure 5:**
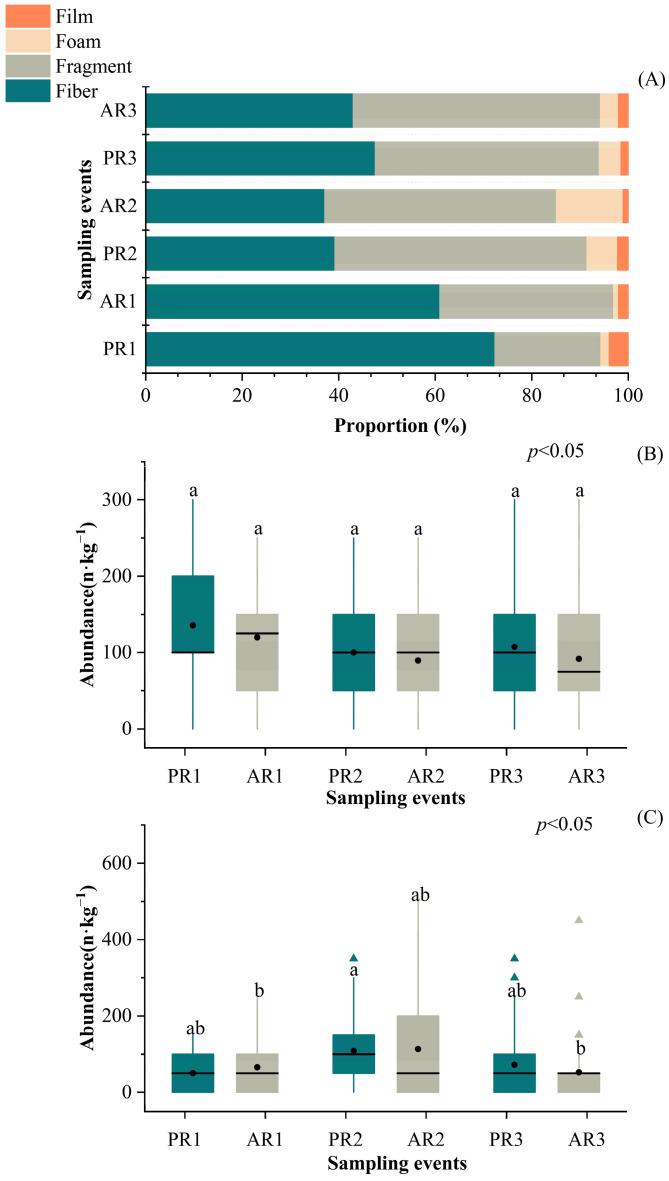
The proportion of microplastics of different shapes (**A**) and the variation in the abundance of fibers (**B**) and fragments (**C**) prior to and after three rainfall events. Note: The lines within boxes indicate medians, the black dots within boxes indicate means, the boxes indicate interquartile intervals, the triangles indicate outliers, and the bars above and below the boxes indicate the upper and lower non-outlier intervals. Different letters represent significant differences among groups at the 95% confidence level.

The proportion of microplastics with different shapes varied among the sampling events. Overall, the dominant shape of microplastics was either fibrous or fragmented for each sampling event. Fibrous particles were more commonly found in PR1, AR1, and PR3, whereas fragmented particles were more prevalent in PR2, AR2, and AR3 (Figure 5A).

The distribution of different-shaped microplastics exhibited different patterns of temporal variation. The abundance of fibrous particles differed significantly among the sampling events (*p* < 0.05). However, there were no significant differences in abundance before and after each rainfall event (*p* > 0.05) (Figure 5B). The abundance of fragments also varied significantly among the sampling events (*p* < 0.05). Significantly lower contamination was observed in AR3 than in PR2 (*p* < 0.05) (Figure 5C). As the foam and film particle samples were inadequate, data analysis was not conducted to assess their variations in this study.

### 3.5. Variations in the Diversity of Microplastics Prior to and after Rainfall Events

**Figure 6:**
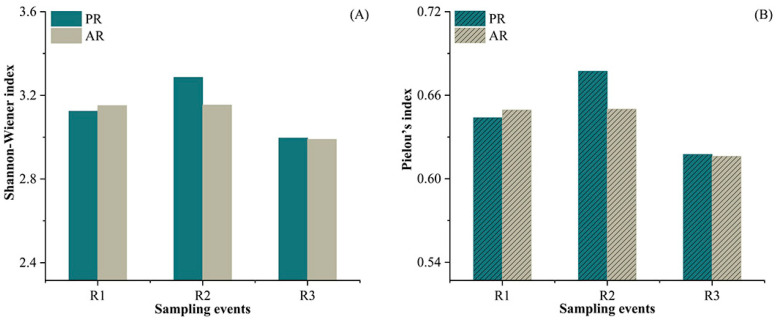
Variation in diversity index of microplastics prior to and after three rainfall events. Data are for all samples from five beaches. Note: (**A**) Shannon–Wiener index; (**B**) Plelou’s index.

Both the Shannon–Wiener index and Pielou’s index were used to estimate the diversity of microplastics in terms of size, color, and shape among the different sampling events (Figure 6). In total, the Shannon–Wiener index decreased in AR3 compared with PR1, with the lowest value at 2.99 (Figure 6A). Similarly, Pielou’s index decreased in AR3 compared with PR1, from 0.64 to 0.62 (Figure 6B). However, there were no significant trends in the variations in the indexes before and after three individual rainfall events (Figure 6A,B). 

### 3.6. Various Responses of Microplastic Concentration to Rainfall Events across Sites

**Figure 7:**
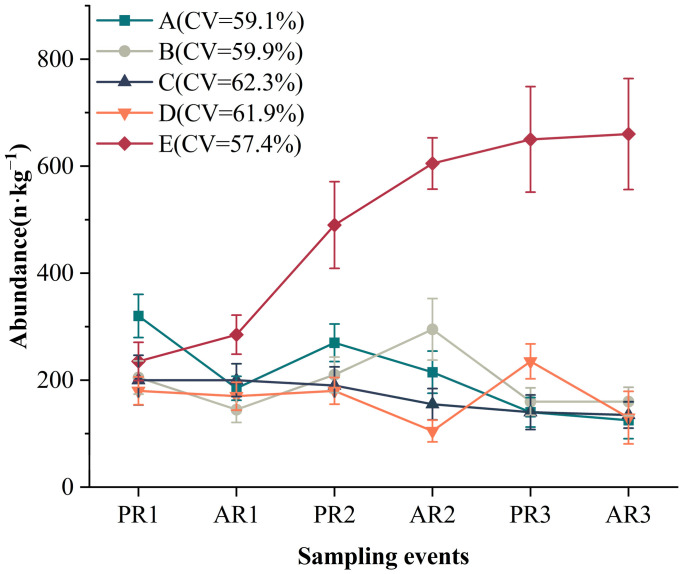
Variations in the abundance of microplastics among rainfall events at different sites (mean ± se). CV represents the coefficient of variation (%) of the abundance of microplastics. The data are for all samples (300) from each event at all sites.

The response of microplastic concentration also varied among sites, with the highest and lowest quantities of 660 ± 103.76 and 105 ± 20.34 n·kg^−1^ detected at site E in AR3 and site D in AR2, respectively. A decreasing trend was observed in the abundance of microplastics at most sites in AR3 compared with PR1, although there were fluctuations in these processes. Moreover, the concentration of microplastics varied the most among the sampling events at site C. In contrast, the abundance of microplastics at site E showed relatively slight variation among the sampling events (Figure 7).

All investigated physicochemical properties of the sediments differed significantly among the sampling sites (*p* < 0.05). The moisture contents of sediments ranged from 6.72% (site A) to 31.97% (site E). For salinity, the maximum was observed at site E (3.8 mS·cm^−1^) and the minimum at site C (2.2 mS·cm^−1^). The median grain sizes of the sediments varied widely at different sites. The median grain size of the sediments at sites C and D was significantly higher than that at the other sites (*p* < 0.05) (Table 2). 

**Figure 8:**
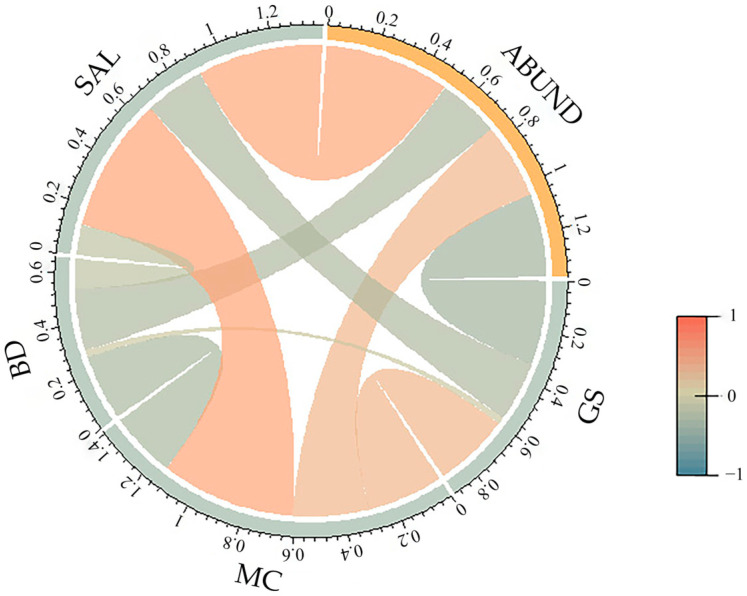
Correlations between the physicochemical properties of the sediments (GS: grain size, MC: moisture content, BD: bulk density, and SAL: pore-water salinity) and the abundance of microplastics (ABUND). Note: The band within the circle represents the correlation between the two variables. The red band indicates a positive correlation, whereas the green band indicates a negative correlation. The intensity of the color and the width of the bands reflect the correlations (more positive or negative).

When the physicochemical properties were examined individually, most were correlated with the distribution of microplastics (Figure 8). The abundance of microplastics was positively correlated with the water content of sediments (*r* = 0.30, *p* < 0.05) and pore-water salinity (*r* = 0.50, *p* < 0.01) and negatively correlated with sediment particle size (*r* = −0.33, *p* < 0.05). No significant relationship was observed between the sediment bulk density and microplastic abundance (*r* = −0.24, *p* > 0.05).

## 4. Discussion

### 4.1. Microplastic Composition on the Beaches of Xiamen City

The concentration of microplastic pollution detected on the beaches of Xiamen was relatively high, surpassing the levels recorded in the beaches of Gujarat State, India (1.4 to 26 n·kg^−1^), Baja California Peninsula, Mexico (135 ± 92 n·kg^−1^), and Tarragona Coast in the Mediterranean (0.7 to 42 n·kg^−1^) [45,46,47]. Located near the Taiwan Strait, Xiamen’s coastal areas may receive microplastics from the South and East China Seas through strait currents. Maritime activities associated with fishing and navigation also contribute to the accumulation of microplastic pollution on coastal beaches [48,49]. Meanwhile, Xiamen stands out as a popular tourist destination, having received 65 million tourists in the last year, which further exposes coastal beaches to elevated levels of plastic contamination (National Bureau of Statistics, Beijing, China, 2023).

Among the detected particles, fibers and fragments were the most common type of microplastic, which is consistent with previous studies [3,50]. Different microplastic shapes tend to represent a variety of pollution sources. Earlier studies have suggested that fibrous microplastics are often released during fishing activities and from clothing, whereas fragmented microplastics originate from the breakdown of large plastic litter [51]. In this study, the high levels of fibrous and fragmented microplastic pollution highlight the impacts of fishing activities and tourism in this area. Blue and black were the dominant colors of microplastics in this study. This result is consistent with the findings for the Baja California Peninsula, Mexico, and Claromecó Beach, Argentina [26,46]. Consistent with previous studies, our study demonstrates that microplastics smaller than 500 μm exhibit the highest pollution levels [3,24,26].

### 4.2. Effects of Rainfall Events on Composition and Diversity of Microplastics on Beaches

Previous studies have demonstrated that the distribution of microplastics on beaches is structured by multiple factors, among which rainfall is crucial [31,33]. However, most studies have focused on variations in microplastic distribution between the rainy and dry seasons, representing a larger temporal scale [32,34]. Building on previous research, this study focused on variations in microplastic abundance and composition during rainfall events on a short-term scale.

In this study, we found that the abundance of microplastics did not differ after an individual rainfall event, but significantly varied after several rainfall events, which is consistent with our first hypothesis. This suggests that a cumulative effect may exist, amplifying the flushing effect of rainwater on microplastics after multiple rainfall events. This finding is similar to that of an earlier study showing that the abundance of microplastics in sediment varies significantly between the neap and spring tide periods, but does not differ on a semidiurnal scale, suggesting that the effects of hydrological processes on microplastic composition take several days to manifest [29]. Alternatively, there is a threshold whereby when rainfall intensity reaches a certain level, the microplastics that may be present on the beach can be washed away. In this study, the intensity was stronger in the last rainfall event than in the previous two events, which may have contributed to the significant decrease in microplastic contamination in the samples of AR3. This is consistent with a previous controlled experiment in which the distribution of microplastics in the soil underwent significant variation as rainfall reached a certain threshold [52]. Previous research has reported the detection of microplastics in rainwater in various areas, such as Toronto, Canada, and Melbourne, Australia [53,54]. However, the concentration of microplastics differed by orders of magnitude between the rainwater and beaches [26,46,53,54]. At present, most studies support the view that rainfall can decrease the concentration of microplastics on beaches, exerting an erosive effect and potentially transporting them into the ocean [33,34]. Physical processes could result in the redistribution of microplastics along coastal areas, potentially reducing pollution concentrations on beaches while simultaneously increasing them in the adjacent seawater [55]. Hence, considering both beaches and adjacent seawater comprehensively to focus on the dynamics of microplastic pollution represents a promising direction for further research. Moreover, to avoid the potential impact of heavy rainfall on the abundance of plastics, field sampling of beaches should be conducted to avoid periods that immediately follow intense rainfall events in the future.

We explored the various response patterns of microplastics in relation to their morphology and size under the influence of rainfall events. Our second hypothesis is also supported. In this study, rainfall events had distinct effects on microplastics of different sizes and shapes. Among the particles, smaller microplastics were more strongly affected by rainfall events, exhibiting a marked decline after multiple rainfall events. However, multiple rainfall events had no clear effect on microplastics larger than 500 μm. This result is consistent with the findings of Zhang et al., who found that microplastics of smaller sizes were more susceptible to the flushing effects of simulated rainfall [56]. These smaller particles could require a lower shear force for activation compared to larger ones [57]. Regarding particles of different shapes, fibrous microplastics exhibited a significant overall variation, whereas fragmented microplastics significantly differed before and after the last two rainfall events. Similar results were observed in a previous study, in which fiber particles in the soil moved farther than foam and film particles during rainfall [56].

As a result, both the Shannon–Wiener index and Pielou’s index decreased after multiple rainfall events. Rainfall events not only alter the composition of microplastics but also further modify their diversity. However, this variation in diversity, similar to abundance, was not significantly evident prior to and after individual rainfall events. This is consistent with the findings within the scope of wetland ecology. During flooding events, flood pulses may introduce other species into the local aquatic community, resulting in an increase in sources. However, persistent and strong hydrodynamics can result in the washout of smaller organisms, ultimately leading to a decrease in diversity [58,59,60]. This study offers a fresh perspective on examining the variations in microplastic composition. In some cases, the diversity of microplastics on beaches may increase, contrary to the findings of this study, under the influence of certain physical processes. It will be necessary to combine the variations in different types of microplastics to identify the shifts in pollution sources.

We also observed that the response of microplastic abundance to rainfall events varied across different locations, which is consistent with our third hypothesis. In this study, the CV of microplastic abundance showed spatial variation among sites. This finding can be explained by the disparities in the beach substrate environment. The physicochemical properties of the sediments were significantly different among the sampling sites in this study. Furthermore, there was a significant correlation between most physicochemical properties and microplastic abundance. The grain size of the sediments was negatively correlated with the abundance of microplastics, indicating that larger sand particles with high porosity may promote the migration of microplastics. We also observed a positive correlation between the moisture content, pore-water salinity, and microplastic concentration at the beaches. This finding is consistent with previous studies conducted in other coastal regions [36,61]. For example, a study conducted in Beibu Gulf, Guangxi, China, revealed that high water content was favorable for microplastics to remain suspended in mobile water-phase pores in mangrove sediments [36]. Thus, clarifying the effects of rainfall events on microplastic composition requires rigorous exploration of the characteristics of the microplastic itself as well as environmental features.

It is essential to note that there may be limitations in accurately identifying smaller microplastic particles in this study due to separation and detection methods. Although NaCl is widely recognized as a readily available and cost-effective solution for density separation, its use may result in low recovery rates of higher-density polymers, potentially leading to underestimations of environmental concentrations. Thus, enhancing the density separation of microplastics could be achieved by employing higher-density solutions like NaI, ZnCl_2_, and ZnBr_2_ [62]. Furthermore, optimizing degradation procedures involves selecting solutions with minimal impact on microplastic particles, such as KOH (10%) [62]. Some research has found that concentrations in field blanks can be higher than those in laboratory blanks, emphasizing the necessity of establishing field blanks to assess potential secondary pollution against field background levels [63]. Thus, we encourage the consideration of improving the procedures in future research.

## 5. Conclusions

This study, for the first time, focused on the effects of rainfall events on the distribution of microplastics on coastal beaches on a short-term scale. Overall, the composition of microplastics showed insignificant variations before and after a single rainfall event, though these variations were amplified after multiple rainfall events. This could be attributed to the cumulative effects of rainfall processes and the threshold effects. When the assemblage of microplastics in the coastal area was viewed as a biological community, the erosion of small particles by rainfall led to a decrease in their diversity. In general, the response of microplastic distribution to rainfall is affected by both objective environmental substrate conditions and the subjective morphology of microplastics. Further studies are needed to replicate this experiment in other coastal areas to develop a more general understanding. We also encourage the consideration of the potential impact of rainfall events during sample collection to ensure the reliability of the data.

## Figures and Tables

**Table 1:** The characteristics of the three selected rainfall events in the rainy season in Xiamen City.

Event	Rainfall Date		Rainfall Duration (h)	Total Rainfall (mm)	Rainfall Intensity (mm·h^−1^)
	Start (yy-mm-dd)	End (yy-mm-dd)			
Rainfall event 1 (R1)	23-04-16	23-04-24	216	66.99	0.31
Rainfall event 2 (R2)	23-05-05	23-05-09	120	36.41	0.30
Rainfall event 3 (R3)	23-06-05	23-06-12	179	73.91	0.41

**Table 2:** Physicochemical properties of sediments at sampling sites, including moisture content, pore-water salinity, and median grain size. Data are median values for six sampling events.

Sites	Moisture Content (%)	Salinity (mS·cm^−1^)	Grain Size (μm)
A	6.72 ^c^	3.1 ^c^	417.19 ^b^
B	7.08 ^c^	3.5 ^c^	398.10 ^b^
C	10.77 ^b^	2.2 ^c^	960.52 ^a^
D	15.62 ^b^	3.7 ^b^	645.26 ^a^
E	31.97 ^a^	3.8 ^a^	426.15 ^b^

Different letters represent significant differences among groups at the 95% confidence level.

## Data Availability

The data presented in this study are available on request from the corresponding author.

## Supplementary Materials

The following supporting information can be downloaded at: https://www.mdpi.com/article/10.3390/toxics12050375/s1, Figure S1: Microscopic images of microplastics: (A) fibers, (B) fragments, (C) foams, and (D) films; Figure S2: FTIR analysis of different microplastic samples; Figure S3: The proportion of polymer types in microplastics of different shapes.
