# Supplementary material for: The Effects of Rainfall Events on the Composition and Diversity of Microplastics on Beaches in Xiamen City on a Short-Term Scale
Source: Toxics. 2024 May 20;12(5):375. doi: 10.3390/toxics12050375 (PMC11125818; doi:10.3390/toxics12050375)
Supplement: Supplementary file 1 [file toxics-12-00375-s001.zip › toxics-3009901-supplementary.pdf]

Supplementary Material

Effects of rainfall events on the composition and diversity of microplastics on beaches in Xiamen City at a short-term scale

Xueyan Li[1] , Fengrun Wu[1, *], Chengyi Zhang[1], Tao Wang[2]

1 School of Environmental Science and Engineering, Xiamen University of Technology, Xiamen 361024, China;
15235771532@163.com (X.L.); zhangchengyi1001@163.com (C.Z.)
2 State Key Laboratory of Estuarine and Coastal Research, East China Normal University, Shanghai 200241, China; warriortao723@gmail.com
*** Correspondence: frwu1993@163.com**

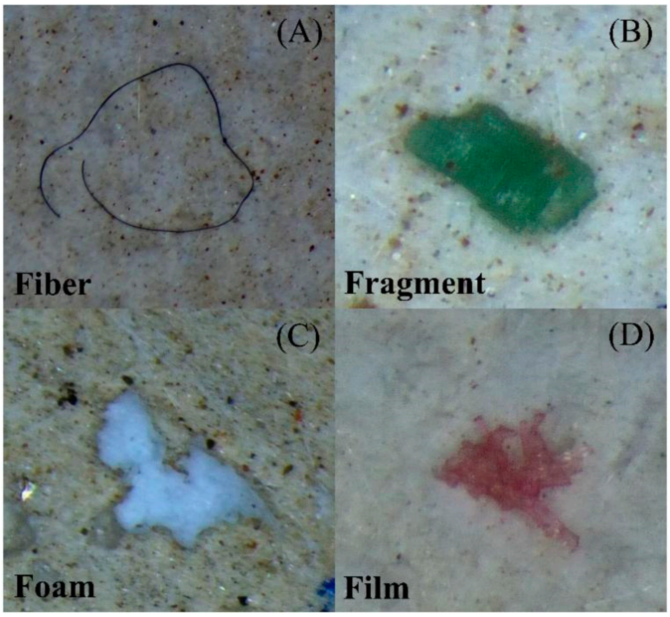

Supplementary Material Figure S1: Microscopic images of microplastics: (A) Fiber, (B) Fragment, (C) Foam, (D) Film.

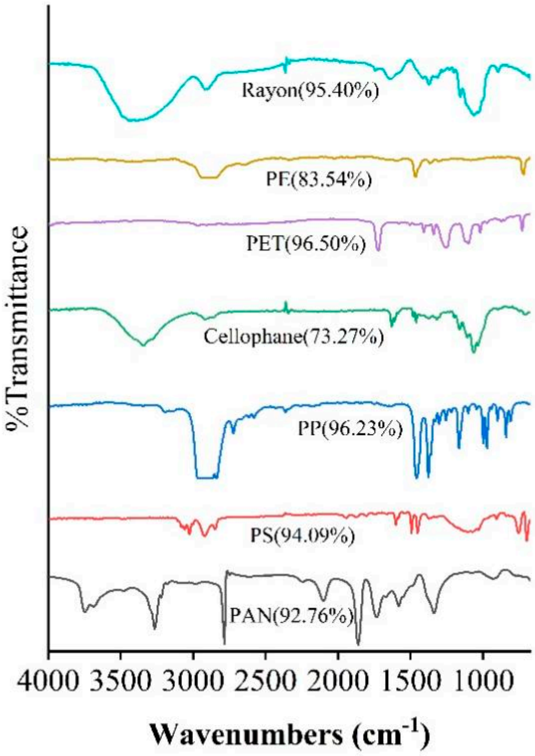

Supplementary Material Figure S2: FTIR spectra of the detected polymer types.

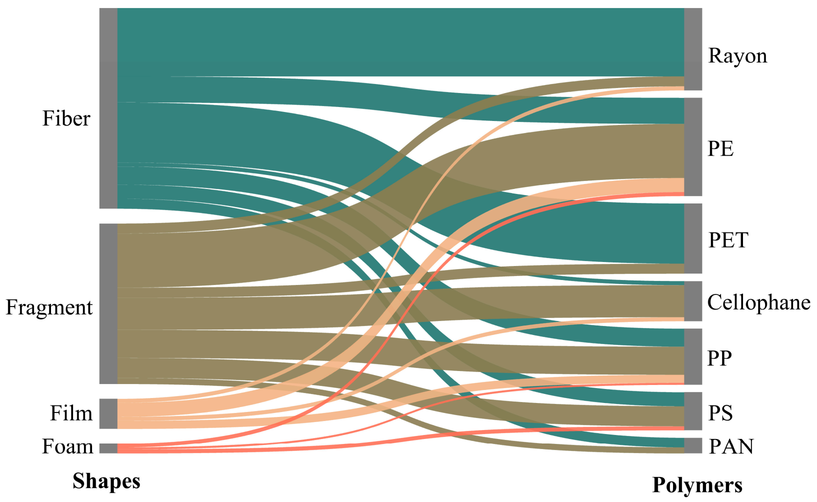

Supplementary Material Figure S3: The proportion of polymer types in microplastics of different shapes.
